# Assessment of psychosocial difficulties by genetic clinicians and distress in women at high risk of breast cancer: a prospective study

**DOI:** 10.1038/s41431-022-01096-9

**Published:** 2022-04-11

**Authors:** Anne Brédart, Jean-Luc Kop, Anja Tüchler, Antoine De Pauw, Alejandra Cano, Julia Dick, Kerstin Rhiem, Peter Devilee, Rita Schmutzler, Dominique Stoppa-Lyonnet, Sylvie Dolbeault

**Affiliations:** 1 Institut Curie, Supportive Care Department, Psycho-oncology Unit, 26 rue d’Ulm, 75005 Paris Cedex 05, France; 2 Psychopathology and Health Process Laboratory UR4057, Psychology Institute, Paris University, 71 Avenue Edouard Vaillant, 92774 Boulogne-Billancourt, France; 3 Université de Lorraine, 2LPN, 3 Place Godefroy de Bouillon, 54015 Nancy Cedex, France; 4 Center for Hereditary Breast and Ovarian Cancer, Faculty of Medicine and University Hospital Cologne, University Hospital of Cologne, University of Cologne, Kerpener Str. 62, 50937 Cologne, Germany; 5 Institut Curie, Department of Genetics, INSERM U830, Paris University, 26 rue d’Ulm, 75005 Paris Cedex 05, France; 6 University Autónoma of Barcelona, Clinical and Health Psychology Department, Barcelona, Spain; 7 Leiden University Medical Centre, Department of Human Genetics, Department of Pathology, S4-P, P.O. Box 9600, 2300 RC Leiden, The Netherlands; 8 CESP, University Paris-Sud, UVSQ, INSERM, University Paris-Saclay, 16 Avenue Paul Vaillant-Couturier, 94807 Villejuif Cedex, France

**Keywords:** Quality of life, Translational research, Patient education

## Abstract

We examined how often genetic clinicians correctly identify psychosocial difficulties in women at high breast cancer risk and explored effects of this assessment and the genetic test result on counselees’ distress. A prospective observational study of counselee–clinician dyads was performed in three French, German and Spanish genetic clinics, involving 709 counselees (participation rate, 83.4%) and 31 clinicians (participation rate, 100%). Counselee–clinician agreement in perceived psychosocial difficulties was measured after the pre-test genetic consultation. Multivariate mixed linear models accounting for clinicians were tested. Predicted distress levels were assessed after the pre- (T1) and post-test result disclosure consultations (T2). Depending on the difficulty domain, clinicians adequately assessed the presence or absence of difficulties in 51% (“familial issues”) to 59% (“emotions”) of counselees. When counselees’ and clinicians’ perceptions disagreed, difficulties were generally underestimated by clinicians. Counselees’ distress levels remained stable from T1 to T2, irrespective of clinicians’ appraisal adequacy, and the genetic test result disclosure. Psychological referral need were found in 20–42% of counselees, more frequently observed for difficulties in the “emotions” domain. Our findings suggest that the genetic test result is a suboptimal indicator for psychological referral. Instead, clinicians should focus on emotions expressed by counselees to appraise their needs for psychological support.

## Introduction

A panel of nine susceptibility genes has been identified as clinically useful for breast cancer risk prediction [1]. Gene panel tests offer important information for making clinical recommendations to women with a personal or familial history of breast cancer. However, with a wider range of high/moderate or lower risk genes being tested and the increased possibility to discover gene variants of uncertain significance (VUS), the information delivered during genetic counseling has become very complex and a source of confusion [2]. Next generation sequencing and the increasing capacity of gene testing also extends the number of women eligible for testing [3], leading to time management challenges for genetic clinicians.

In routine practice, gene panel testing is firstly proposed to a woman in the family who developed cancer (index case) to identify a possible breast cancer genetic factor. If a pathogenic variant is found, blood relatives are proposed targeted single gene testing to identify or rule out the presence of the known pathogenic variant identified in the family. They are also increasingly offered gene panel testing [4]. Following gene panel testing, a pathogenic variant, a VUS or a negative uninformative result, which does not explain the personal or familial cancer history, may be obtained. Following targeted single gene testing, result may reveal either the presence or absence of a pathogenic variant.

Women with a personal or familial history of breast cancer who ask for genetic testing (i.e., the “counselees”) often experience psychosocial difficulties in relation to genetic testing relating to: informed choice (i.e., to choose between screening or risk reducing surgery), understanding the cancer risks associated with the result (e.g., appraisal of risks, coping with a result of unknown clinical significance), and the personal and familial consequences of the test [5]. These difficulties may demand particular attention and deserve specific support.

Limited adverse psychological outcomes have been observed following the disclosure of the gene test result [6]. Carriers of a pathogenic variant experienced higher short-term distress after testing [7] which remits after 1 year [8]; higher distress was also reported when a VUS had been identified [6, 9]. However, some counselees, particularly those who feel unprepared or lack of support, may experience distress [10]. Adequate identification of counselees who might require additional genetic counseling and support would help minimize distress [11].

The major part of the cancer genetic consultation is usually devoted to the provision of biomedical information, leaving less time to enquire about counselees’ concerns associated with genetic testing [12]. Moreover, although emotional factors are strong predictors of distress after genetic testing [7], psychological referral is more often based on the genetic test result than on distress [13]. This may result in unfulfilled psychosocial care needs during the course of genetic testing [14].

Because of these concerns, attention is increasingly devoted to clinician–counselee communication in genetic testing [15]. In the broader oncology field, initiative such as the completion of psychosocial questionnaires before routine consultation seems to facilitate discussion about patients’ psychosocial concerns during the consultation, which positively affects physicians’ awareness and management of patients’ problems and health outcomes [16]. Similarly, in the cancer genetic consultation, adequate appraisal of counselees’ psychosocial difficulties would also positively affect counselees by eliciting genetic clinicians’ action such as providing extra counseling or support, psychosocial information or referral to psychosocial services and so lowering their distress.

To date, only one study performed in a single country has documented the genetic clinicians’ little awareness of counselees’ psychosocial difficulties in the context of familial cancer genetic testing [14]. Moreover, that study was not designed to clarify which domains of psychosocial problems needed improved counselors’ awareness to affect counselees’ distress level.

We designed this prospective observational study to evaluate the extent to which genetic clinicians correctly identify domains of psychosocial difficulties in counselees at the initial (pre-test) consultation for breast and ovarian cancer genetic susceptibility testing. The study also examined the effect of clinicians’ appraisals of these difficulty domains on counselees’ distress after the initial consultation and after the genetic test result disclosure. Lastly, the study assessed the effect of disclosure of the genetic test result on counselees’ distress.

## Methods

The study protocol was approved in France by the Comité consultatif sur le traitement de l’information en matière de recherche dans le domaine de la santé (CCTIRS: Consultative committee for information management in health research—No. 16.314) and the Comité de Protection des Personnes Ile-de-France V (CPP—No. 18.12.28.38743 CAT2), in Germany by the Ethics Committee of the University Hospital of Cologne (No. 16-098) and in Spain by the Ethics Committee of the Instituto Catalán de Oncología of Barcelona (No. PR111/16). All recruited women provided written informed consent.

### Study design

This prospective observational study was part of a multicenter psychosocial research [17] undertaken within the European research program BRIDGES (“Breast Cancer Risk after Diagnostic Gene Sequencing”) (https://bridges-research.eu). It comprised two successive cohorts of consecutive female counselees undergoing testing for breast cancer risk, one using current national breast cancer gene panel [18, 19] (cohort 1—November 2016–April 2018) and the other using the BRIDGES (TruRisk® v3.1.1 [20]) gene panel (cohort 2—November 2019–December 2020).

### Counselee–clinician dyads

Counselees comprised women aged 18 years or over, with a personal and/or familial history of breast cancer, who were consecutively approached on the day of the initial (pre-test) visit. All clinicians in the genetic clinics of Curie Institute (France), University Hospital of Cologne (Germany) and Catalan Institute of Oncology (Spain) participated in the study.

Counselee–clinician dyads were obtained for four samples from the two successive cohorts constituted within BRIDGES. The first and second samples comprised women with a personal (index cases) or familial history of breast cancer. Index cases received diagnostic breast cancer gene panel testing; women free of cancer were offered predictive targeted testing. These women were approached at Curie Institute (France, sample 1) and the Catalan Institute of Oncology (Spain, sample 2) [17]. The third and fourth samples involved women free of a personal breast cancer who underwent either predictive targeted testing and the gene panel testing implemented within BRIDGES [20] if a pathogenic variant had been identified in the family, or otherwise only gene panel testing. These women were approached at Curie Institute (sample 3) and the Center for Hereditary Breast and Ovarian Cancer at the University Hospital Cologne (Germany, sample 4).

Counselees with a new, recurrent or advanced breast cancer (in the first and second samples), another type of cancer or a major psychiatric disorder were not included in the study.

Counselees who agreed to participate were invited to complete the study questionnaires at home (online or on paper) within 2 weeks after the pre-test consultation (T1) and again within 2 months after the post-test consultation (T2). When necessary, a reminder call was made to counselees. Questionnaires not completed within 1 month afterwards were considered missing. Clinicians completed questionnaires within 1 week after the pre-test consultation.

### Questionnaires and data collection

Socio-demographic and clinical data were collected from counselees after the pre-test consultation and from medical records.

**Fig. 1:**
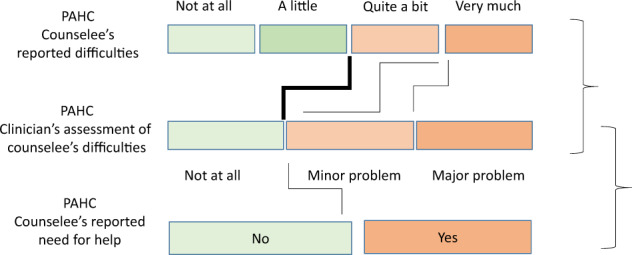
Agreement calculation. Thick line indicates cut-offs for the counselees’ and clinicians’ PAHC response scale which were selected as they were optimal in terms of positive predictive value.

The extent of genetic-specific psychosocial difficulties was assessed at T1 using the 26-item “Psychosocial Aspects of Hereditary Cancer” (PAHC) questionnaire [5] available in French, German and Spanish [21]. Items are grouped into six conceptually defined domains [5]: hereditary predisposition, practical, familial, emotional, living with cancer and children-related issues (Supplementary Table S2 provides examples of items). Possible answers are: Not at all (1), A little (2), Quite a bit (3) and Very much (4). The presence of a difficulty in a domain is determined according to possible cut-offs to an item response scale as shown in Fig. 1. A difficulty was considered present if a counselee indicated “quite a bit”, or “very much” on at least one item within a given difficulty domain, or alternatively, a response “very much”. For each domain, counselees are also asked about their need for additional help (Yes vs. No).

Counselees’ self-reported distress was measured by French [22], German [23] and Spanish [24] versions of the Hospital Anxiety and Depression Scale (HADS) at T1 and at T2. HADS total scores range from 0 to 42.

The PAHC questionnaire filled out by the clinician independently from counselees examines the presence and severity of problem in the same domains as those of the PAHC counselees’ version on a 3-level rating scale [5]: No problem (1), Minor problem (2) and Important problem (3).

### Statistical analyses

A sample size of at least 500 counselees was predicted to allow for multivariate analyses.

Statistical analyses of dyad agreement were restricted to questions that were answered by both counselees and clinicians.

We assessed rates of agreement for counselee–clinician dyads using different possible cut-offs of the counselee and clinician response scales as shown in Fig. 1. The thick line in Fig. 1 indicates the selected cut-offs; using these cut-offs, a counselee was considered as presenting difficulty in a domain if she answered “Quite a bit” or “Very much” to at least one item in that domain and the clinician was considered as judging the presence of a problem if he/she reported a “minor” or “major” problem. These cut-offs were chosen as they maximized the positive predictive value of clinicians’ assessment (i.e., the probability of agreement on the presence of difficulties) as this reflects an appropriate referral of counselee in a context of limited time and resources in psychological support (see Supplementary Table S3 for detailed data by cut-off).

For each counselee–clinician dyad and PAHC domain, we calculated proportions of responses where counselees and clinicians agreed (true positives (TP) and true negatives (TN)), and where clinicians under- (false negatives (FN)) or overestimated (false positives (FP)) counselees’ difficulties. The level of agreement between counselees and clinicians was statistically quantified using the Kappa statistics with confidence intervals (CI) [25].

The Kappa statistic provides a quantitative measure of the magnitude of agreement between counselees and clinicians. Kappa statistic ranges from −1 (total disagreement) to +1 (perfect agreement). Kappa values from 0.01 to 0.20, 0.21 to 0.40, and above 0.41 equate to slight, fair, and moderate to substantial agreement [25]. A Kappa of zero and a 95% confidence of Kappa statistics including zero indicates no agreement at all.

**Fig. 2:**
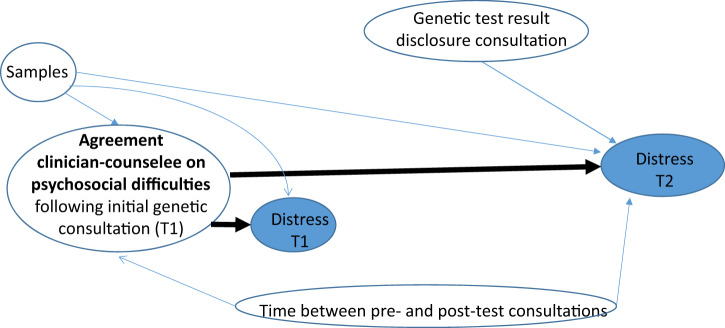
Hypotheses on the effect of agreement between clinicians and counselees on distress at T1 and T2, and factors susceptible to influence this effect. The thick arrows represent the effect being tested. T1 = within 1 month after the initial genetic consultation; T2 = within 3 months after the genetic test disclosure consultation. The theoretical model presumes that **a** “sample” would influence “agreement” at T1 and “distress” at T1 and T2; **b** that the effect of “agreement” at T1 on distress at T2 would depend on time elapsed between T1 and T2; **c** the influence of distress at T1 on distress at T2 was not considered because distress at T1 is part of the link between “agreement” at T1 and distress at T2; **d** genetic test result at T2 is independent from “agreement” and distress at T1; the influence of this factor on distress at T2 is tested independently.

The effect of dyad agreement (i.e., TP, FP or FN vs. TN) on distress measured by the HADS at T1 and at T2 was tested according to theoretical assumptions on relationships between factors deemed to influence this effect [6–8] (Fig. 2). To this end, multivariable mixed linear models were fitted taking the TN category as reference. The basic model comprised the intercept, the random effect of clinicians on the intercept and the fixed effect of samples. For distress at T1, the basic model was compared to: (1) a first model including the effect of agreement, and (2) a second model in which the interaction between agreement and samples was added to the first model. For distress at T2, the basic model was compared to: (1) a first model including the effect of agreement, (2) a second model based on the first model plus time lapse between the pre- and post-test consultations and its interaction with agreement, and (3) a third model based on the second model plus genetic test result (pathogenic variant, negative vs. uninformative results) and its interaction with agreement. Model selection was based on the Bayesian information criterion (BIC) [26]. Beta coefficients derived from the selected model were used for predicting HADS means and 95% CI for each TN, TP, FP, and FN agreement category. The effect of inter-clinicians’ variability on the outcome was assessed by intra-class correlation coefficients (ICC).

**Fig. 3:**
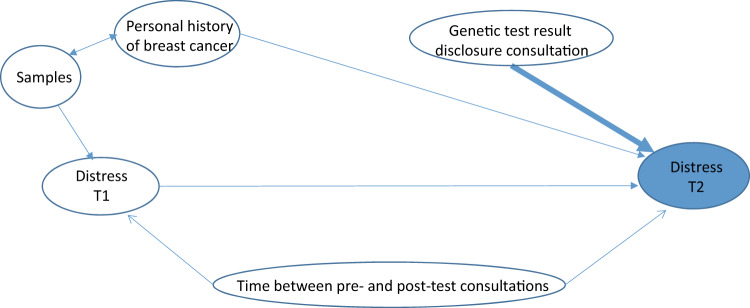
Hypotheses on the effect of genetic test results on distress at T2, and factors susceptible to influence this effect. The thick arrow represents the effect being tested by the study. T1 = within 1 month after the initial genetic consultation; T2 = within 3 months after the genetic test disclosure consultation. The theoretical model presumes that **a** distress at T2 would depend on distress at T1; **b** a personal history of breast cancer would influence distress associated with the disclosure of the genetic test result at T2; **c** “sample” would influence “distress” at T2 via the personal history of breast cancer; **d** the effect of “distress” at T1 on “distress” at T2 would depend on the time elapsed between T1 and T2.

Another set of multivariable mixed linear models examined the effect of the genetic test result on counselees’ distress at T2. Because of the marked difference in personal breast cancer history [7], separate models were fitted for samples 1 and 2, and for samples 3 and 4. Moreover, VUS were communicated to counselees in sample 1 and 2, but not in samples 3 and 4. The effect of the genetic test result on distress was tested according to theoretical assumptions on relationships between factors deemed to influence this effect (Fig. 3). The basic model comprised the intercept, the random effect of clinicians on the intercept and the fixed effect of samples. The basic model was compared to: (1) a first model including HADS at T1, (2) a second model based on the first model plus time lapse between the pre- and post-test consultations, (3) a full model based on the second model plus genetic test results. For samples 1 and 2, addition of the presence or absence of personal breast cancer was added to the first, second and third models. Model selection was based on the BIC.

Statistical analyses were performed with R software (R Core Team, 2020).

## Results

A total of 850 eligible counselees were consecutively approached, 709 of which (83.4%) agreed to participate (Table 1). Eligible counselees and non-participants at T1 or at T2 did not significantly differ by age, having children or, where applicable, disease status and genetic test result of the first person tested in the family, except in sample 3 where participants at T1 were slightly older.

**Table 1:** Counselees’ socio-demographic and clinical characteristics (*N* = 850).

	Sample 1—FR1 *N* = 258	Sample 2—SP *N* = 170	Sample 3—FR2 *N* = 200	Sample 4—GE *N* = 222
Age Mean (SD)	48.0 (12.1)	47.8 (12.8)	39.3 (13.3)^a^	41.0 (9.9)
Age Median (range)	47.5 (21–82)	47.8 (19–81)	36 (21–80)	41 (21–71)
Having children Yes *n* (%)	203 (78.7)	114 (77.0)	97 (48.5)	140 (63.1)
Clinical data				
Personal history of breast cancer *n* (%) (Yes)	208 (80.6)	108 (63.9)	NA	NA
Genetic test result in first person tested in the family *n* (%)				
Pathogenic variant	34 (13.2)	49 (28.8)	200 (100)	54 (25.0)
Non-informative result	NA	NA	NA	97 (44.9)
Variant of uncertain significance	NA	NA	NA	4 (1.9)
First person tested in the family not accessible	NA	NA	NA	37 (17.1)
First person eligible for testing in the family not yet tested	NA	NA	NA	24 (11.1)
Respondents at T1	*N* = 213	*N* = 133	*N* = 157	*N* = 206
Education level *n* (%)				
Compulsory education or below	6 (2.8)	57 (33.3)	4 (2.6)	8 (3.9)
Secondary or technical/vocational education	60 (28.4)	55 (32.6)	36 (23.4)	117 (56.8)
Higher education or above	145 (68.7)	58 (34.1)	114 (74.0)	81 (39.3)
Marital status *n* (%)				
Married/partnered	149 (70.3)	102 (77.3)	94 (60.3)	146 (70.9)
Others (widowed, separated/ divorced, single/never married)	63 (29.7)	30 (22.7)	62 (39.7)	60 (29.1)
Loss of family member because of breast/ovarian cancer *n* (%) (Yes)	86 (42.8)	60 (46.9)	76 (49.0)	122 (59.2)
Past psychological help *n* (%) (Yes)	113 (53.1)	51 (38.3)	73 (47.1)	87 (42.2)
Respondents at T1 and T2	Sample 1—FR1 *N* = 164	Sample 2—SP *N* = 67	Sample 3—FR2 *N* = 118	Sample 4—GE *N* = 199
Time lapse between pre- and post-test consultations (days) Mean (SD)	163 (17)	100 (57)	108 (17)	97 (33)
Number of counselees with BRCA1, BRCA2 vs. other high and moderate-risk pathogenic variant	24/1	16/0	31/2	17/14
Number of counselees with a negative result	14	14	85	24
Number of counselees with an uninformative vs. VUS^b^	113/12	23/14	0	143

*FR* France, *GE* Germany, *SP* Spain, *T1* within 1 month after the pre-test consultation, *T2* within 3 months after the post-test consultation, *NA* not applicable either because no index case tested yet (samples 1 and 2) or counselee’s eligibility only if family member carrier of a pathogenic variant (sample 3).

^a^Comparisons between eligible counselees and respondents at T1 and at T2; participant in sample 3 at T1 are older (mean (SD) age = 40.3 (13.2); *p* value = <0.05).

^b^VUS = variant of uncertain clinical significance; VUS not communicated to counselees in samples 3 and 4.

Counselee–clinician dyads were constituted for 213 counselee respondents in sample 1 (France), 133 in sample 2 (Spain), 157 in sample 3 (France), and 206 in sample 4 (Germany).

Among eligible counselees, 208 (80.6%) in sample 1 and 108 (63.9%) in sample 2 had a personal breast cancer history. In sample 3 and 4, 200 (100%) and 54 (25%) counselees had a family member carrier of a breast cancer pathogenic variant.

Among counselees’ respondents, 25 (sample 1), 16 (sample 2), 33 (sample 3) and 31 (sample 4) were informed they were carrying a pathogenic variant. Supplementary Table S4 details comparisons of the four samples’ characteristics.

All 31 clinicians (6 of them being the same in samples 1 and 3) involved in genetic counseling in the three clinics participated in the study (Supplementary Table S1). Clinicians were mostly female (86%), with variable experience in genetic counseling. Among them, 5 of 10, and 8 of 12 were clinical geneticists with a medical background in sample 1 and 3, respectively; all 4 clinicians were genetic counselors in sample 2, and 10 of 11 clinicians were gynecologists with a genetic training in sample 4. Clinicians did not differ by country on age, gender and length of clinical experience.

### Counselee–clinician agreement

Less than 10% and 11% missing data were observed for counselees’ and clinicians’ individual items respectively, except for children-related issues, which had up to 36% and 25% missing data for counselees’ and clinicians’ items, respectively (Supplementary Table S3b). For all domains, answers from both counselees and clinicians were available for 94% of dyads. An exception was the domain of children-related issues with 67% dyads. For the different PAHC scales, the overall number of TN, TP, FP and FN assessments by clinicians were 170, 207, 126, 164 (Hereditary predisposition), 302, 71, 156, 137 (Practical issues), 162, 178, 123, 202 (Familial issues), 233, 160, 157, 116 (Emotions), 26, 323, 29, 284 (Living with cancer), and 72, 173, 59, 172 (Children-related-issues) (Supplementary Table S3).

Overall, comparable proportions of difficulties were reported by counselees and clinicians for the first four domains (Table 2). Counselee–clinician agreement per domain ranged from 51.1% (familial issues) to 59.0% (emotions). Disagreement between counselees and clinicians varied across samples and domains. In samples 1, 2 and 3, underestimation was more pronounced than overestimation, whereas the reverse was observed for sample 4. Underestimation was particularly marked in all four samples for the domain of “living with cancer”. In this domain, 91.5% of counselees reported difficulties, but 53.1% of clinicians did; most differences originated from the systematic underestimation of difficulties by clinicians, which resulted in the low negative predictive value of 7% (i.e., TN/(TN + FN)) (see Supplementary Table S3 for details). Agreement between clinicians and counselees for the domain of “emotions” ranged from 59.5 to 64.8% in samples 1, 2 and 3. Underestimation by clinicians was generally less marked in this domain.

**Table 2:** Assessment of genetic-specific psychosocial difficulties by counselees and by clinicians.

PAHC domains^a^	Counselees’ responses “Quite a bit”/“Very much”	Clinicians’ responses “Minor”/“Important”	Agreement rate ^b^ (true positives and negatives)	Underestimation^b^ (false negatives)	Overestimation^b^ (false positives)	Agreement level Kappa (95% CI)
Hereditary predisposition	388 (55.0)	335 (50.0)	377 (56.5)	164 (24.6)	126 (18.9)	0.13 (0.05–0.21)
Sample 1 (FR1)	113 (53.1)	82 (41.6)	112 (56.9)	55 (27.9)	30 (15.2)	0.15 (0.02–0.28)
Sample 2 (SP)	86 (65.2)	51 (38.4)	78 (59.1)	45 (34.1)	9 (6.8)	0.24 (0.10–0.38)
Sample 3 (FR2)	89 (57.1)	66 (42.3)	91 (58.7)	43 (27.7)	21 (13.5)	0.19 (0.05–0.34)
Sample 4 (GE)	100 (48.8)	136 (73.9)	96 (52.5)	21 (11.5)	66 (36.1)	0.06 (−0.07–0.18)
Practical issues	218 (30.9)	228 (34.0)	373 (56.0)	137 (20.6)	156 (23.4)	0.001 (−0.08–0.08)
Sample 1 (FR1)	79 (37.1)	32 (16.2)	118 (59.6)	60 (30.3)	20 (10.1)	0.01 (−0.11–0.13)
Sample 2 (SP)	59 (45.4)	36 (27.1)	78 (60.0)	38 (29.2)	14 (10.8)	0.16 (0.01–0.32)
Sample 3 (FR2)	45 (28.8)	54 (34.8)	92 (59.7)	26 (16.9)	36 (23.4)	0.08 (−0.08–0.24)
Sample 4 (GE)	35 (17.0)	106 (57.6)	85 (46.2)	13 (7.1)	86 (46.7)	0.02 (−0.08–0.12)
Familial issues	399 (56.7)	304(45.4)	340 (51.1)	202 (30.4)	123 (18.5)	0.04 (−0.04–0.11)
Sample 1 (FR1)	145 (68.7)	73 (37.1)	101 (51.8)	78 (40.0)	16 (8.2)	0.12 (0.01–0.23)
Sample 2 (SP)	79 (60.3)	58 (43.6)	70 (53.4)	42 (32.1)	19 (14.5)	0.10 −0.06–0.26
Sample 3 (FR2)	103 (66.0)	54 (34.6)	83 (53.6)	60 (38.7)	12 (7.7)	0.15 (0.03–0.28)
Sample 4 (GE)	72 (35.0)	119 (64.7)	86 (46.7)	22 (12.0)	76 (41.3)	0.02 (−0.10–0.14)
Emotions	288 (40.9)	220 (47.7)	393 (59.0)	116 (17.4)	157 (23.6)	0.17 (0.09–0.25)
Sample 1 (FR1)	96 (45.5)	69 (34.8)	127 (64.8)	46 (23.5)	23 (11.7)	0.28 (0.15–0.41)
Sample 2 (SP)	52 (39.7)	62 (46.6)	78 (59.5)	22 (16.8)	31 (23.7)	0.18 (0.01–0.35)
Sample 3 (FR2)	75 (48.1)	75 (48.1)	100 (64.5)	27 (17.4)	28 (18.1)	0.29 (0.14–0.44)
Sample 4 (GE)	65 (31.6)	114 (62.0)	88 (47.8)	21 (11.4)	75 (40.8)	0.04 (−0.08–0.16)
Living with cancer	643 (91.5)	355 (53.1)	349 (52.7)	284 (42.9)	29 (4.4)	0.002 (−0.04–0.05)
Sample 1 (FR1)	197 (93.4)	83 (42.3)	85 (43.8)	105 (54.1)	4 (2.1)	0.02 (−0.04–0.08)
Sample 2 (SP)	129 (98.5)	63 (47.4)	62 (47.3)	68 (51.9)	1 (0.8)	−0.002 (−0.04–0.04)
Sample 3 (FR2)	133 (85.8)	67 (43.2)	73 (47.7)	72 (47.1)	8 (5.2)	0.04 (−0.06–0.14)
Sample 4 (GE)	184 (89.3)	142 (77.2)	129 (70.1)	39 (21.2)	16 (8.7)	−0.05 (−0.17–0.06)
Children-related issues	369 (70.6)	285 (47.1)	245 (51.5)	172 (36.1)	59 (12.4)	0.04 (−0.04–0.12)
Sample 1 (FR1)	142 (78.9)	81 (46.6)	83 (52.2)	63 (39.6)	13 (8.2)	0.06 (−0.06–0.18)
Sample 2 (SP)	92 (92.0)	49 (37.7)	45 (45.0)	53 (53.0)	2 (2.0)	0.05 (−0.04–0.13)
Sample 3 (FR2)	63 (63.6)	35 (29.4)	55 (61.1)	31 (34.4)	4 (4.4)	0.29 (−0.13–0.44)
Sample 4 (GE)	72 (50.0)	120 (65.9)	62 (48.8)	25 (19.7)	40 (31.5)	−0.04 (−0.21–0.13)

Table entries are number (%) of counselees, except column of concordance level.

*CI* confidence interval.

^a^Genetic-specific difficulties assessed with the Psychosocial Aspects in Hereditary Cancer (PAHC) questionnaire covering six domains of difficulties.

^b^Agreement, under- and overestimation based on data available for both counselees and clinicians.

The kappa statistic showed no or only slight agreement between counselees’ and clinicians’ perception of difficulties across all PAHC domains with Kappa statistics (95% CI) ranging from 0.001 (−0.08–0.08) (practical issues) to 0.17 (0.09–0.25) (emotions). No agreement (95% CI including 0) was found for “practical issues”, “familial issues”, “living with cancer”, and “children-related issues”. Kappa was fair (>0.20) for “hereditary predisposition” (sample 2), “emotions” (samples 1 and 3) and “children-related issues” (sample 3).

### Effect of counselee–clinician agreement on distress after pre- and post-test consultations

HADS showed fairly stable distress levels from T1 to T2 (Table 3). Proportions of women with HADS scores above the threshold at which psychological referral is recommended (>12) [27], ranged from 19.9 to 42.2% across the four samples.

**Table 3:** Counselees’ HADS means (95% CI) and number (%) of counselees with HADS scores >12 at T1 and at T2 by sample.

Psychosocial scale or parameter (range of values)	Sample 1—FR1	Sample 2—SP	Sample 3—FR2	Sample 4—GE
Number of counselees at T1	213	133	157	206
Hospital Anxiety and Depression Scale at T1 (HADS) [0–42]—Distress^a,b^	11.6 (10.8–12.4)	10.0 (8.8–11.2)	10.7 (9.8–11.7)	8.5 (7.6–9.5)
Number (%) of counselees with HADS score >12 at T1	89 (42.2)	43 (32.8)	52 (32.5)	41 (19.9)
Number of counselees at T2	164	67	118	199
Hospital Anxiety and Depression Scale at T2 (HADS) [0–42]—Distress^a,c^	11.4 (10.5–12.3)	9.1 (7.3–10.9)	10.5 (9.4–11.6)	7.5 (6.5–8.6)
Number (%) of counselees with HADS score >12 at T2	64 (37.4)	19 (28.4)	44 (34.4)	43 (21.5)

Across samples for the HADS and assessment times (T1 and T2), <5 missing data were observed and internal consistencies (Cronbach’s alpha coefficients) are very good all above 0.80.

Comparisons between samples: ^a^(sample 1 vs. sample 4), *p* < 0.0001; ^b^(sample 3 vs. sample 4): *p* value = <0.05, <0.01; ^c^(sample 3 vs. sample 4): *p* value = <0.01

Of the various statistical models that were fitted with distress as outcome, the lowest BIC at both T1 and T2 was obtained for models that included dyad agreement (i.e., TP, FP, FN vs. TN) and samples (1 to 4) in five of the six PAHC domains: for example, to predict distress at T1 and testing agreement in the “Hereditary predisposition” domain, the statistical models that included either samples (basic model), or sample and agreement (first model), or the interaction between samples and agreement (second model) resulted in BIC estimates of 4417.451, 4382.435 (i.e., lower BIC, first model) and 4432.558, respectively; to predict distress at T2, the statistical models that included either samples (basic model), or samples and agreement (first model), or samples, agreement and the interaction between agreement and time between T1 and T2 (second model), or sample, agreement and the interaction between the genetic test result and agreement (third model) resulted in BIC estimates of 3417.414, 3399.314 (i.e., lower BIC, first model), 3419.991, 3436.978, respectively (see Supplementary Tables S5 and S6 showing similar results for other PAHC domains except “Living with Cancer” for which samples (basic model) best explained data.).

Length of time between the pre- (T1) and post-test (T2) consultations did not improve the statistical model fit according to the BIC parameter.

Table 4 provides predicted mean of HADS and 95% CI at T1 (second column) and at T2 (third column) for counselees for which clinicians’ assessments were TN, TP, overestimation (FP) or underestimation (FN) for the different PAHC domains. It shows that mean HADS scores and 95% CI in the four agreement categories remained comparable from T1 to T2. For all PAHC domains, clinicians’ adequate perception of low amount of difficulties (TN) as well as overestimation of difficulties (FP) at both T1 and at T2 were associated with HADS scores ranging from 6.6 to 9.4. In contrast, clinicians’ adequate perception (TP) of greater amount of difficulties as well as underestimation (FN) were associated with significantly higher HADS scores (*p* < 0.001), ranging from 9.6 to 14.8. Differences in HADS scores for the domain “living with cancer” were less consistent owing to the small number of counselees in the TN and FP categories.

**Table 4:** HADS predicted means (95% confidence interval) for distress after the pre-test (T1) and after the post-test (T2) consultations according to counselee–clinician agreement in perceived genetic-specific psychosocial difficulties in statistical models best fitting the data.

	Distress at T1	Distress at T2
PREDICTOR (number of counselees by categories of counselee–clinician agreement)	HADS predicted means (95% CI)	HADS predicted means (95% CI)
PAHC hereditary predisposition		
True negative (*n* = 170)	7.7 (6.7–8.7)	7.0 (5.7–8.2)
True positive (*n* = 207)	12.1 (11.2–13.0)**	11.2 (10.1–12.4)**
Overestimation (*n* = 126)	9.0 (7.8–10.2)	8.8 (7.4–10.3)*
Underestimation (*n* = 164)	11.3 (10.3–12.3)**	11.0 (9.7–12.2)**
PAHC practical issues		
True negative (*n* = 302)	8.9 (8.1–9.8)	8.4 (7.4–9.5)
True positive (*n* = 71)	12.6 (11.1–14.1)**	12.6 (10.7–14.6)**
Overestimation (*n* = 156)	9.4 (8.3–10.6)	8.5 (7.2–9.9)
Underestimation (*n* = 137)	12.7 (11.6–13.9)**	11.7 (10.3–13.0)**
PAHC familial issues		
True negative (*n* = 162)	7.7 (6.7–8.7)	7.3 (6.0–8.6)
True positive (*n* = 178)	12.7 (11.7–13.6)**	12.0 (10.7–13.2)**
Overestimation (*n* = 123)	7.4 (6.1–8.6)	6.6 (5.1–810)
Underestimation (*n* = 202)	11.7 (10.8–12.7)**	11.4 (10.1–12.6)**
PAHC emotions		
True negative (*n* = 233)	7.4 (6.6–8.2)	7.1 (6.0–8.1)
True positive (*n* = 160)	14.8 (13.9–15.7)**	13.6 (12.5–14.8)**
Overestimation (*n* = 157)	7.4 (6.5–8.4)	7.4 (6.2–8.7)
Underestimation (*n* = 116)	13.3 (12.2–14.3)**	11.9 (10.5–13.2)**
PAHC living with cancer		
True negative (*n* = 26)	6.6 (4.01–9.09)	7.1 (4.0–10.2)
True positive (*n* = 323)	11.0 (10.2–11.8)**	10.1 (9.0–11.1)
Overestimation (*n* = 29)	7.4 (5.0–9.4)	7.1 (4.3–10.0)
Underestimation (*n* = 284)	10.0 (9.1–10.8)*	9.6 (8.4–10.7)
PAHC children-related issues		
True negative (*n* = 72)	7.8 (6.3–9.4)	7.3 (5.3–9.3)
True positive (*n* = 173)	11.7 (10.7–12.8)**	11.9 (10.5–13.3)**
Overestimation (*n* = 59)	6.7 (4.9–8.5)	6.7 (4.5–8.8)
Underestimation (*n* = 172)	10.9 (9.8–12.0)**	10.4 (8.9–11.8)**

T1 = within 1 month after the pre-test consultation; T2 = within 3 months after the post-test consultation. Table entries refer to predicted mean values of HADS and 95% confidence interval at T1 (second column) and at T2 (third column) for counselees for which clinicians’ assessments were true negative, true positive, overestimation (false positive) or underestimation (false negative) for the different PAHC domains. Statistical significance tests taking “True negative” in agreement as the reference category. Best models selected based on Bayesian information criterion (BIC) estimates includes the random effect of clinicians, samples and agreement, except for the PAHC domain “living with cancer” where the basic model is best.

*FR* France, *GE* Germany, *SP* Spain.

*,**p values <0.05; <0.001.

The largest difference between categories in HADS scores were observed for the PAHC “emotions” domain. Five to seven point-differences in HADS scores were noticeable between the TP or FP categories, and the TN or FN categories. Meanwhile in other domains, differences between these pairs of categories did not exceed five-point scores. Moreover, the lower CI limits of mean HADS scores in the TP of the “emotions” domain exceeded 12 at T1 and T2. The picture was nearly equivalent for underestimation. Hence for the “emotions” domain, most counselees falling in the TP and FN categories experienced anxious or mood symptoms associated with a HADS scores above 12.

The modest inter-clinicians’ variability on HADS scores (ICC of 0.04 or below in retained models) indicates similar distress levels between counselees across clinicians.

### Effect of genetic test results

The communication of the genetic test result, i.e., a pathogenic variant on a high breast cancer susceptibility gene such BRCA1 or BRCA2, a negative or an uninformative test result, or a VUS (samples 1 and 2) had no significant influence on distress measured after the post-test consultation: HADS predicted means (95% CI) ranged from 8.8 (6.9–10.8) (negative result) to 10.8 (10.0–12.0) (uninformative result) at T1 and from 8.3 (7.2–9.4) (pathogenic variant) to 10.0 (8.4–10.9) (uninformative result) at T2 (Supplementary Tables S7 and S8).

## Discussion

This large prospective observational study in the context of breast cancer genetic testing in three European clinical settings is the first to address the ability of genetic clinicians to identify counselees with psychosocial difficulties.

Agreement rates between psychosocial difficulties reported by counselees and clinicians’ ratings of these difficulties were generally low, although better than rates reported by previous oncology (non-genetic) studies (i.e., 26–38% (health information) and 28–40% (psychological difficulties) [28, 29] compared to 51–59% in this study). However, clinicians did under-estimate difficulties in up to 42.9% of counselees.

As the role endorsed by genetic clinicians places greater emphasis on biomedical information [30], we expected that their ability to identify counselees’ difficulties would be noticeable in the domain of hereditary predisposition (e.g., counselees worrying about the chance of being a carrier of a genetic mutation). However, this was only found in the Spanish sample.

Difficulties in the “emotions” domain were better identified, especially by clinicians from the French setting (65%). Emotions may be more easily recognizable [31, 32] than difficulties in other domains which may require more in-depth evaluation of the personal and familial context.

Distress scores were low on average across samples. However, around 20–42% of counselees displayed distress levels eligible for psychological referral. Regardless of samples, time between pre- and post-test consultations and genetic test results, counselee–clinician agreement on psychosocial difficulties best explained distress levels after consultations. However, counselees’ distress remained stable between the pre- and post-test consultations and this appeared independent from clinicians’ adequate or inadequate perception of the presence or absence of difficulties. Counselees experienced higher and persisting distress levels when their difficulties were underestimated or when they were correctly identified, suggesting that both improving clinicians’ appraisals and their response to counselees’ difficulties in terms of psychological referral should be promoted. Accurate appraisal of the presence of difficulties by clinicians was not associated to lower distress levels, suggesting a lack of clear clinical pathways between detection of emotional difficulties and intervention in these cancer genetic clinical setting [11].

Furthermore, all counselees considered together, distress seemed to remain unaffected by the disclosure of the genetic test result. This counterintuitive finding has already been observed in other cancer genetics studies [6, 33, 34]. It suggests that psychological referral should not rely on the genetic test result criterion only.

Genetic counseling is meant to minimize distress experienced by counselees [35]. However, addressing psychological concerns is rarely on the genetic consultations agenda [36]. In our study, a psychological consultation was proposed to counselees depending on clinician’s personal judgment.

As mentioned in the introduction, a randomized trial in the Netherlands tested the usefulness of the PAHC questionnaire in routine practice [14]. This questionnaire was completed by counselees allocated to the intervention group before the consultation but not by the control group. After evaluating the PAHC responses, clinicians undertook initiatives to discuss overall psychosocial difficulties with counselees. However, this intervention reduced counselees’ distress levels, by −1.4 (95% CI: 0.2–2.6) point of mean HADS, almost negligible compared to the 4 to 7 average difference in mean HADS between counselees in the TN vs. FN categories found in our study. Of note, a two-point score difference is the minimal clinically important difference in HADS scores [37].

In contrast, this study found large differences (>5-point HADS) in distress levels between counselees with more and less difficulties for the PAHC “emotions” domain. Feasible approaches may be suggested to help clinicians appraise the severe difficulties requiring psychological referral, such as questions focusing on emotions to be completed by counselees before the pre-test consultation, and multidisciplinary team discussions involving psychological experts to ensure appropriate psychological care.

### Strengths and limitations

The present study has limitations. Generalization is limited as samples were constituted of counselees’ willing to be tested, from only one genetic clinic per country, at specific time points of the cancer genetic testing trajectory (after the pre- and post-test consultations). Samples 1 and 3 comprised mostly highly educated women. However, sample biases were accounted for in statistical models. Moreover, in contrast to other studies [29, 38], levels of agreement were similar across clinicians regardless of samples and their cultural and health care delivery differences.

Collection of distress data before the initial consultation was not possible as women could have been sensitized by the cancer genetic context at any time before the study took place. Because clinicians had variable encounters with counselees with different personal and familial history, we could not explore in depth the relationships between clinicians’ characteristics and agreement. Counselee–clinician verbal and non-verbal interactions during the consultation should be investigated to understand communication factors underlying agreement or disagreement in perceived psychosocial difficulties [39].

This study rests on a large sample of participants, including all clinicians of each genetic clinic, a high rate of respondents among counselees, and little differences between respondents and non-respondents. It was performed in three European country settings, reflecting different genetic testing practices. The study included counselees routinely met in cancer genetic clinics, either for predictive or diagnostic genetic testing for breast cancer, and is particularly relevant considering the rapidly evolving cancer genetic testing field.

To conclude, these findings suggest that referral to psychological assessment and support should not be based on the genetic test result criterion. Enhancing assessment of emotional difficulties particularly would provide more relevant guidance for appraising psychological needs. This could be achieved through the systematic implementation of questions focusing on emotions to be answered by counselees before the pre-test consultation, and by multidisciplinary team discussions involving psychological experts.

## Supplementary information

Supplementary material

## Data Availability

The study database is hosted at Institut Curie (France). It may be available after main publication for BRIDGES research program performed (in 2023).

## Code availability

The data generated and analyzed during this study can found within the article and its Supplementary files. Additional data are available from the corresponding author on reasonable request.
