# Supplementary material for: Assessment of psychosocial difficulties by genetic clinicians and distress in women at high risk of breast cancer: a prospective study
Source: Eur J Hum Genet. 2022 Apr 11;30(9):1067–75. doi: 10.1038/s41431-022-01096-9 (PMC9437045; doi:10.1038/s41431-022-01096-9)
Supplement: Supplementary file 1 — Supplementary material [file 41431_2022_1096_MOESM1_ESM.docx]

**Supplementary material figure S1. Agreement calculation.**

Note. Thick line indicates the optimal cut-off for the counselees’ and clinicians’ PAHC response scale in terms of positive predictive value.

**
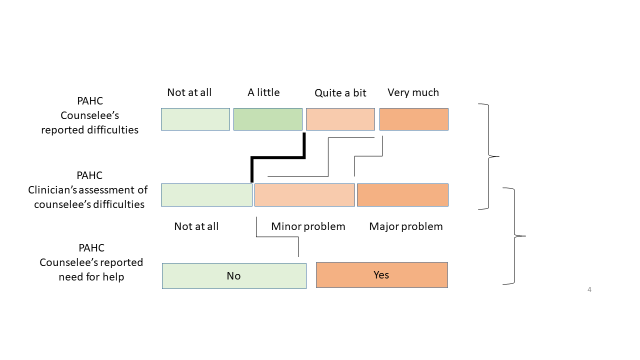
**

**Supplementary material figure S2. Hypotheses on the effect of agreement between clinicians and counselees on distress at T1 and T2, and factors susceptible to influence this effect.**

Note: The thick arrows represent the effect being tested. T1 = within 1 month after the initial genetic consultation; T2 = within 3 months after the genetic test disclosure consultation. The theoretical model presumes that (a) “sample” would influence “agreement” at T1 and “distress” at T1 and T2; (b) that the effect of “agreement” at T1 on distress at T2 would depend on time elapsed between T1 and T2; (c) the influence of distress at T1 on distress at T2 was not considered because distress at T1 is part of the link between “agreement” at T1 and distress at T2; (d) genetic test result at T2 is independent from “agreement” and distress at T1; the influence of this factor on distress at T2 is tested independently.


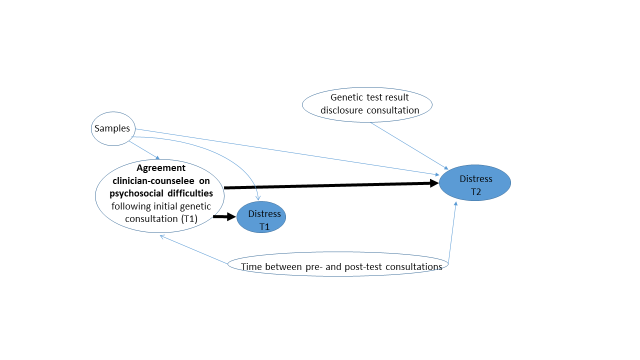
**Supplementary material figure S3. Hypotheses on the effect of genetic test results on distress at T2, and factors susceptible to influence this effect.**

Note: The thick arrow represents the effect being tested by the study. T1=within 1 month after the initial genetic consultation; T2=within 3 months after the genetic test disclosure consultation. The theoretical model presumes that (a) distress at T2 would depend on distress at T1; (b) a personal history of breast cancer would influence distress associated with the disclosure of the genetic test result at T2; (c) “sample” would influence “distress” at T2 via the personal history of breast cancer; (d) the effect of “distress” at T1 on “distress” at T2 would depend on the time elapsed between T1 and T2.


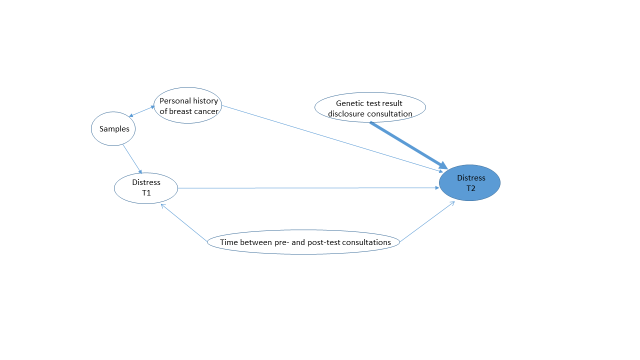


**Supplementary material table S1. Clinicians’ characteristics**

|  | **Sample 1**  **(N=10)** | **Sample 2**  **(N=4)** | **Sample 3**  **(N=12, including 6 clinicians from sample 1)** | **Sample 4**  **(N=11)** |
| --- | --- | --- | --- | --- |
| **Age (Years)** |  |  |  |  |
| 20-29 | 1 | 0 | 0 | 0 |
| 30-39 | 4 | 1 | 5 | 4 |
| 40-49 | 1 | 2 | 2 | 5 |
| 50-59 | 2 | 1 | 3 | 1 |
| 60 & + | 2 | 0 | 2 | 1 |
| **Gender –** Female (Yes) | 8 | 4 | 10 | 10 |
| **Professional background** |  |  |  |  |
| Genetic counselor (biology background) | 5 | 1 | 8 | 0 |
| Genetic counselor (nurse) | 0 | 3 | 0 | 0 |
| Clinical geneticist (medical background) | 5 | 0 | 3 | 1 |
| Gynecologist (genetic training) | 0 | 0 | 1 | 10 |
| **Years of experience in cancer genetics (Years)** |  |  |  |  |
| 1-5 | 4 | 1 | 4 | 4 |
| 6-10 | 0 | 0 | 0 | 3 |
| 11-15 | 3 | 1 | 4 | 1 |
| 16-20 | 0 | 0 | 0 | 3 |
| 21+ | 3 | 2 | 4 | 0 |
| **Genetic clinic location** | France | Spain | France | Germany |
| **Data collection period** | 2016-18 | | 2019-20 | |

Note: Six clinicians in France participated in cohorts 2016-18 2019-20. Clinicians met a number of counselees from 1 to 61, 9 to 60, 1 to 35 and 2 to 49, in sample 1, 2, 3 and 4, respectively. Of all counselees, clinicians aged less than 40 years old met 81.6%, 11.3%, 52.9% and 37.6% of them, in sample 1, sample 2, sample 3 and sample 4, respectively, which was significantly different (p < .001).

| **Supplementary material table S2. Prevalence of counselees’ response “quite a bit”/”very much” to the ‘Psychosocial Aspects in Hereditary Cancer’ (PAHC) questionnaire by item (%) and to at least one item per PAHC dimension, and by sample N (%) after the initial genetic consultation.**   \| **Psychosocial Aspects in Hereditary Cancer (PAHC)** \| **Sample 1 – FR1**  **(N=213)** \| **Sample 2 - SP**  **(N=133)** \| **Sample 3 – FR2**  **(N=157)** \| **Sample 4 - GE**  **(N=206)** \| \| --- \| --- \| --- \| --- \| --- \| \| **PAHC items by domain** \|  \|  \|  \|  \| \| **Hereditary predisposition ^@^** \| 113 (53.1%) \| 86 (65.2%) \| 89 (57.1%) \| 100 (48.8%) \| \| Worried about the chance of being a carrier of a genetic mutation ^@@^ \| 40.1% \| 52.3% \| 42.6% \| 32.2% \| \| Worried about having to choose whether or not to go for genetic counselling and testing ^@@@, &&&&, ###^ \| 3.8% \| 25.8% \| 7.2% \| 8.6% \| \| Worried about the choice of possible preventive options (screening or surgery) ^@, &&&^ \| 21.5% \| 41.7% \| 29.8% \| 26.5% \| \| Worried about coping with the (future) DNA test results ^@@@, $, &&^ \| 24.1% \| 42.3% \| 32.7% \| 19.7% \| \| Worried about (fulfilling) your plans for having children \| 15.9% \| 13.7% \| 24.5% \| 22.3% \| \| ***Need for help (Yes)* ^@@@@,^** ^§§, &, ##^ \| 22.2% \| 37.2% \| 17.5% \| 9.2% \| \| **Practical issues ^@@@@, §§§§, $, #^** \| 79 (37.1%) \| 59 (45.4%) \| 45 (28.8%) \| 35 (17.0%) \| \| Worried about the impact of genetic testing on your daily life ^@@@@, §, $$, &&&, ##^ \| 21.4% \| 43.1% \| 24.4% \| 10.7% \| \| Worried about the impact of genetic testing on obtaining insurance or mortgage ^&&&, ***, §§§§^ \| 27.8% \| 10% \| 12.3% \| 8.3% \| \| ***Need for help (Yes) ^@@@@,^*** ^§§, &&, ###^ \| 14.1% \| 29.2% \| 9.0% \| 4.9% \| \| **Familial issues ^@@@@, §§§§, $$$$^** \| 145 (68.7%) \| 79 (60.3%) \| 103 (66.0%) \| 72 (35.0%) \| \| Misunderstood by partner/family/social circle with respect to genetic testing \| 3.4% \| 4.6% \| 5.1% \| 3.9% \| \| Bothered by lack of support about genetic testing from partner, family or your social circle \| 4.3% \| 3.1% \| 3.2% \| 0.5% \| \| Worried about immediate family’s functioning because of genetic testing ^$^ \| 8.3% \| 6.2% \| 11.6% \| 3.5% \| \| Worried about the contact with family members about genetic testing \| 9.5% \| 7% \| 10.9% \| 4.4% \| \| Worried about coping with cancer within the family ^@@@@, §§§§, $$$$^ \| 60.3% \| 56.5% \| 61.0% \| 29.0% \| \| Burdened by feelings of responsibility towards family members related to genetic testing ^§§§§,$$$$, &&&&^ \| 39.7% \| 17.8% \| 30.3% \| 10.6% \| \| ***Need for help (Yes)*** *^@@@@,^* ^§§§^ \| 19.6% \| 25.6% \| 13.5% \| 6.3% \| \| **Emotions** ^§, $$^ \| 96 (45.5%) \| 52 (39.7%) \| 75 (48.1%) \| 65 (31.6%) \| \| Anxious ^@, §§§§,$$$^ \| 26.1% \| 19.2% \| 25.0% \| 8.3% \| \| Tense \| 20.6% \| 18% \| 22.4% \| 16.1% \| \| Depressed \| 8.2% \| 5.5% \| 12.2% \| 7.3% \| \| Insecure about the future ^@@, §§§,$$^ \| 27.3% \| 25.4% \| 25.3% \| 11.2% \| \| Concerned about life and death ^@@,§^ \| 30.9% \| 34.6% \| 29.5% \| 18.4% \| \| ***Need for help (Yes) ^@@@@,^***^§§§, ##^ \| 21.5% \| 33.8% \| 16.8% \| 8.3% \| \| **Living with cancer** ^@@@, ###^ \| 197 (93.4%) \| 129 (98.5%) \| 133 (85.8%) \| 184 (89.3%) \| \| Emotionally burdened that family members have cancer ^@@@@, §§§§,$$$, &&&&, ####^ \| 71.6% \| 96.2% \| 68.5% \| 47.5% \| \| Emotionally burdened by losing a family member because of cancer ^@@, &&&&, ##^ \| 83.1% \| 97.7% \| 85.4% \| 88.5% \| \| Emotionally burdened by your diagnostic or treatment of cancer \| 51.7% \| 49.6% \| 57.6% \| / \| \| Worried about the chance of getting cancer (again) ^@, §§§^ \| 65.6% \| 60.2% \| 56.5% \| 45.1% \| \| Worried about the chance that family members will get cancer ^@@@@, §, &&&, ###^ \| 71.9% \| 89.3% \| 69.9% \| 58.5% \| \| ***Need for help (Yes)*** *^@@@@,^* ^§§§§,$, ##^ \| 29.4% \| 39.2% \| 19.2% \| 8.7% \| \| **Children-related issues** ^@@@@, §§§§, &&, ####, *^ \| 142 (78.9%) \| 92 (92%) \| 63 (63.6%) \| 72 (50.0%) \| \| Guilty about the chance of passing possible genetic alterations on to children^@@@@, §§§§, $$^ \| 39.1% \| 47% \| 29.5% \| 11.1% \| \| Worried about telling children the results ^@@@@, §§§, ##^ \| 29.0% \| 43% \| 19.4% \| 10.6% \| \| Worried about the chance of children developing cancer ^@@@@, §§§§, &&, ####, *^ \| 76.4% \| 92% \| 61.1% \| 48.6% \| \| ***Need for help (Yes)*** *^@@@@,^* ^§§§§,&, ####, ***^ \| 26.6% \| 42.4% \| 8.7% \| 6.5% \|   Comparisons between samples: & (sample 1 vs sample 2); * (sample 1 vs sample 3); § (sample 1 vs sample 4); # (sample 2 vs sample 3); @ (sample 2 vs sample 4); $ (sample 3 vs sample 4): p value= <0.05, <0.01, <.0001, <0.0001 by number of symbol.  **Supplementary material table S3: Diagnostic performance of clinicians’ assessment by cut-off on the counselee and clinician PAHC response scales** | | | | | | | | | |
| --- | --- | --- | --- | --- | --- | --- | --- | --- | --- | --- | --- | --- | --- | --- | --- | --- | --- | --- | --- | --- | --- | --- | --- | --- | --- | --- | --- | --- | --- | --- | --- | --- | --- | --- | --- | --- | --- | --- | --- | --- | --- | --- | --- | --- | --- | --- | --- | --- | --- | --- | --- | --- | --- | --- | --- | --- | --- | --- | --- | --- | --- | --- | --- | --- | --- | --- | --- | --- | --- | --- | --- | --- | --- | --- | --- | --- | --- | --- | --- | --- | --- | --- | --- | --- | --- | --- | --- | --- | --- | --- | --- | --- | --- | --- | --- | --- | --- | --- | --- | --- | --- | --- | --- | --- | --- | --- | --- | --- | --- | --- | --- | --- | --- | --- | --- | --- | --- | --- | --- | --- | --- | --- | --- | --- | --- | --- | --- | --- | --- | --- | --- | --- | --- | --- | --- | --- | --- | --- | --- | --- | --- | --- | --- | --- | --- | --- | --- | --- | --- | --- | --- | --- | --- | --- | --- | --- | --- | --- | --- | --- | --- | --- | --- | --- | --- | --- | --- | --- | --- | --- | --- | --- | --- | --- | --- | --- | --- | --- | --- | --- | --- | --- | --- | --- | --- | --- | --- | --- | --- | --- | --- | --- | --- | --- | --- | --- | --- | --- | --- | --- | --- | --- | --- | --- | --- | --- | --- | --- | --- |
| **PAHC domains** | **True negative**  **(Negative agreement)**  **N** | **True positive**  **(Positive agreement)**  **N** | **False negative (Underestimation)**  **N** | **False positive (Overestimation)**  **N** | **Agreement level**  **Kappa** | **Sensitivity** | **Specificity** | **Positive predictive value** | **Negative predictive value** |
| ***Counselees’ response ‘Very much’ vs clinicians’ response ‘Important’*** |  |  |  |  |  |  |  |  |  |
| Hereditary Predisposition | 452 | 14 | 189 | 12 | 0.06 | 0.07 | 0.97 | 0.54 | 0.71 |
| Practical issues | 576 | 3 | 78 | 9 | 0.03 | 0.04 | 0.98 | 0.25 | 0.98 |
| Familial issues | 452 | 17 | 186 | 10 | 0.08 | 0.08 | 0.98 | 0.63 | 0.71 |
| Emotions | 527 | 12 | 110 | 17 | 0.10 | 0.10 | 0.97 | 0.41 | 0.83 |
| Living with cancer | 179 | 35 | 444 | 4 | 0.03 | 0.07 | 0.98 | 0.90 | 0.29 |
| Children-related issues | 231 | 19 | 216 | 10 | 0.04 | 0.08 | 0.96 | 0.66 | 0.52 |
| ***Counselees’ response ‘Quite a bit or Very much’ vs clinicians’ response ‘Minor or Important’*** |  |  |  |  |  |  |  |  |  |
| Hereditary Predisposition | 170 | 207 | 164 | 126 | 0.13 | 0.56 | 0.57 | 0.62 | 0.51 |
| Practical issues | 302 | 71 | 137 | 156 | 0.00 | 0.34 | 0.66 | 0.31 | 0.68 |
| Familial issues | 162 | 178 | 202 | 123 | 0.04 | 0.47 | 0.57 | 0.59 | 0.45 |
| Emotions | 233 | 160 | 116 | 157 | 0.17 | 0.58 | 0.60 | 0.51 | 0.67 |
| Living with cancer | 26 | 323 | 284 | 29 | 0.00 | 0.53 | 0.47 | 0.92 | 0.08 |
| Children-related issues | 72 | 173 | 172 | 59 | 0.04 | 0.50 | 0.55 | 0.75 | 0.30 |
| ***Counselees’ response ‘Very much’ vs clinicians’ response ‘Minor/Important’*** |  |  |  |  |  |  |  |  |  |
| Hereditary Predisposition | 258 | 127 | 76 | 206 | 0.15 | 0.63 | 0.56 | 0.38 | 0.77 |
| Practical issues | 385 | 27 | 54 | 200 | -0.01 | 0.33 | 0.66 | 0.12 | 0.88 |
| Familial issues | 263 | 102 | 101 | 199 | 0.06 | 0.50 | 0.57 | 0.34 | 0.72 |
| Emotions | 307 | 80 | 42 | 237 | 0.14 | 0.66 | 0.56 | 0.25 | 0.88 |
| Living with cancer | 98 | 267 | 212 | 85 | 0.07 | 0.56 | 0.54 | 0.76 | 0.32 |
| Children-related issues | 126 | 117 | 118 | 115 | 0.02 | 0.50 | 0.52 | 0.50 | 0.52 |
| ***Counselees’ response ‘Yes, need for help’ vs clinicians’ response ‘Minor/Important’*** |  |  |  |  |  |  |  |  |  |
| Hereditary Predisposition | 271 | 79 | 57 | 250 | 0.07 | 0.58 | 0.52 | 0.24 | 0.83 |
| Practical issues | 369 | 30 | 58 | 194 | -0.00 | 0.34 | 0.66 | 0.13 | 0.86 |
| Familial issues | 308 | 55 | 50 | 242 | 0.05 | 0.52 | 0.56 | 0.19 | 0.86 |
| Emotions | 290 | 73 | 52 | 238 | 0.09 | 0.58 | 0.55 | 0.23 | 0.85 |
| Living with cancer | 232 | 80 | 69 | 267 | 0.00 | 0.54 | 0.46 | 0.23 | 0.77 |
| Children-related issues | 209 | 58 | 51 | 205 | 0.02 | 0.53 | 0.51 | 0.22 | 0.80 |

| **Supplementary material table S3b. Missing data to the PAHC items** |  |  |  |  |
| --- | --- | --- | --- | --- |
| ***Counselees (difficulties)*** | **Sample 1** | **Sample 2** | **Sample 3** | **Sample 4** |
| PAHC Hereditary predisposition | 0 (0) | 1 (1) | 1 (1) | 1 (0) |
| PAHC Practical issues | 0 (0) | 3 (2) | 1 (1) | 0 (0) |
| PAHC Familial issues | 2 (1) | 2 (2) | 1 (1) | 0 (0) |
| PAHC Emotions | 2 (1) | 2 (2) | 1 (1) | 0 (0) |
| PAHC Living with cancer | 2 (1) | 2 (2) | 2 (1) | 0 (0) |
| PAHC Children-related issues | 33 (15) | 33 (25) | 58 (37) | 62 (30) |
| ***Counselees (wish for help)*** |  |  |  |  |
| PAHC Hereditary predisposition | 6 (3) | 4 (3) | 3 (2) | 0 (0) |
| PAHC Practical issues | 14 (7) | 3 (2) | 2 (1) | 0 (0) |
| PAHC Familial issues | 9 (4) | 4 (3) | 3 (2) | 0 (0) |
| PAHC Emotions | 13 (6) | 3 (2) | 3 (2) | 0 (0) |
| PAHC Living with cancer | 12 (6) | 3 (2) | 6 (4) | 0 (0) |
| PAHC Children-related issues | 25 (12) | 34 (26) | 56 (36) | 7 (3) |
| ***Clinicians*** | **Sample 1** | **Sample 2** | **Sample 3** | **Sample 4** |
| PAHC Hereditary predisposition | 16 (8) | 0 (0) | 1 (1) | 22 (11) |
| PAHC Practical issues | 15 (7) | 0 (0) | 2 (1) | 22 (11) |
| PAHC Familial issues | 16 (8) | 0 (0) | 1 (1) | 22 (11) |
| PAHC Emotions | 15 (7) | 0 (0) | 1 (1) | 22 (11) |
| PAHC Living with cancer | 17 (8) | 0 (0) | 2 (1) | 22 (11) |
| PAHC Children-related issues | 39 (18) | 3 (2) | 39 (25) | 24 (12) |

**Supplementary material S4. Significant differences between samples on socio-demographic and clinical characteristics**

Comparisons between samples:

Age=significant difference between Sample 1 & Sample 3; Sample 1 & Sample 4; Sample 2 & Sample 3; Sample 2 & Sample 4 at p value<0.0001;

Having children= significant difference between Samples overall at p value<0.0001;

Being affected with BC=significant difference between Sample 1 & Sample 2 at p value<0.0001;

Genetic test result of first person tested in the family=significant difference between Sample 3 & Sample 4 at p value<0.0001.

For respondents:

Education level= significant difference between Samples overall at p value<0.0001;

Marital status= significant difference between Samples overall at p value<0.05;

Loss family member= significant difference between Samples overall at p value<0.01;

Past psychological help=significant difference between Samples overall at p value<0.05;

**Supplementary material table S5. Estimates for distress after the pre-test (T1) consultation according to mixed linear models including samples and counselee-clinician agreement in perceived genetic-specific psychosocial difficulties.***

| **Model:** | **Basic** | **1** | **2** | **BIC** | **Deviance** | **p value** | **ICC** | **Marginal R2/Conditional R2** |
| --- | --- | --- | --- | --- | --- | --- | --- | --- |
| **Outcome: HADS at T1** |  |  |  |  |  |  |  |  |
| **PAHC domain: Hereditary predisposition** |  |  |  |  |  |  |  |  |
| **Variables** |  |  |  |  |  |  |  |  |
| Samples | X | X | X | 4417.451 | 4378.443 | - | 0.00 | 0.035 / 0.039 |
| Agreement |  | X | X | 4382.435 | 4323.924 | <0.000 | 0.00 | 0.109 / 0.113 |
| Agreement * samples |  |  | X | 4432.558 | 4315.534 | 0.50 | 0.01 | 0.119 / 0.123 |
| **PAHC domain: Practical issues** |  |  |  |  |  |  |  |  |
| **Variables** |  |  |  |  |  |  |  |  |
| Samples | X | X | X | 4416.372 | 4377.374 | - | 0.00 | 0.037 / 0.039 |
| Agreement |  | X | X | 4391.968 | 4333.470 | <0.000 | 0.01 | 0.098 / 0.103 |
| Agreement * samples |  |  | X | 4435.719 | 4318.723 | 0.10 | 0.00 | 0.116 / 0.118 |
| **PAHC domain: Familial issues** |  |  |  |  |  |  |  |  |
| **Variables** |  |  |  |  |  |  |  |  |
| Samples | X | X | X | 4411.637 | 4372.647 | - | 0.00 | 0.035 / 0.037 |
| Agreement |  | X | X | 4348.535 | 4290.050 | <0.000 | 0.00 | 0.147 / 0.149 |
| Agreement * samples |  |  | X | 4404.303 | 4287.334 | 0.98 | 0.00 | 0.148 / 0.149 |
| **PAHC domain: Emotions** |  |  |  |  |  |  |  |  |
| **Variables** |  |  |  |  |  |  |  |  |
| Samples | X | X | X | 4417.244 | 4378.245 | - | 0.00 | 0.035 / 0.037 |
| Agreement |  | X | X | 4234.073 | 4175.574 | <0.000 | 0.00 | 0.287 / 0.287 |
| Agreement * samples |  |  | X | 4287.718 | 4170.722 | 0.85 | 0.00 | 0.289 / 0.289 |
| **PAHC domain: Living with cancer** |  |  |  |  |  |  |  |  |
| **Variables** |  |  |  |  |  |  |  |  |
| Samples | X | X | X | 4399.362 | 4360.390 | - | 0.00 | 0.035 / 0.037 |
| Agreement |  | X | X | 4400.768 | 4342.311 | <0.001 | 0.00 | 0.061 / 0.061 |
| Agreement * samples |  |  | X | 4445.622 | 4328.707 | 0.14 | 0.00 | 0.079 / 0.081 |
| **PAHC domain: Children-related issues** |  |  |  |  |  |  |  |  |
| **Variables** |  |  |  |  |  |  |  |  |
| Samples | X | X | X | 3158.200 | 3121.208 | - | 0.01 | 0.056 / 0.063 |
| Agreement |  | X | X | 3140.577 | 3085.088 | <0.0001 | 0.02 | 0.125 / 0.140 |
| Agreement * samples |  |  | X | 3189.760 | 3078.782 | 0.71 | 0.02 | 0.134 / 0.149 |

* For distress at T1, the basic model with the intercept, random effect of clinicians on the intercept, and the fixed effects of samples is compared to: 1) a first model including the effect of agreement, and 2) a second model adding the interaction between agreement and samples. The best model is selected based on Bayesian Information Criterion (BIC) estimates. Marginal R2 provides the variance explained only by fixed effects and conditional R2 provides the variance explained by the entire model, i.e., both fixed effects and random effects. Intra-class correlation coefficient (ICC) informs on the clinician variability effect. Statistical significance tests take ‘True negative’ as the reference category. *, ***=p values < 0.05; 0.001.

**Supplementary material table S6. Estimates for distress after the post-test (T2) consultation according to mixed linear models including samples, counselee-clinician agreement in perceived genetic-specific psychosocial difficulties, time T1 to T2 and the genetic test result.***

| **Model:** | **Basic** | **1** | **2** | **3** | **BIC** | **Deviance** | **p value** | **ICC** | **Marginal R2/Conditional R2** |
| --- | --- | --- | --- | --- | --- | --- | --- | --- | --- |
| **Outcome: HADS at T2** |  |  |  |  |  |  |  |  |  |
| **PAHC domain: Hereditary predisposition** |  |  |  |  |  |  |  |  |  |
| **Variables** |  |  |  |  |  |  |  |  |  |
| Samples | X | X | X | X | 3417.414 | 3380.031 | - | 0.02 | 0.052 / 0.073 |
| Agreement |  | X | X | X | 3399.314 | 3343.239 | <0.0001 | 0.01 | 0.115 / 0.127 |
| Time T1 to T2 & Time * agreement |  |  | X | X | 3419.991 | 3338.994 | 0.37 | 0.01 | 0.122 / 0.135 |
| Genetic test results (PV, N, UN) & genetic test result * agreement |  |  |  | X | 3436.978 | 3331.060 | 0.09 | 0.02 | 0.135 / 0.155 |
| **PAHC domain: Practical issues** |  |  |  |  |  |  |  |  |  |
| **Variables** |  |  |  |  |  |  |  |  |  |
| Samples | X | X | X | X | 3430.448 | 3393.041 | - | 0.02 | 0.054 / 0.074 |
| Agreement |  | X | X | X | 3419.412 | 3363.302 | <0.0001 | 0.02 | 0.105 / 0.120 |
| Time T1 to T2 & Time * agreement |  |  | X | X | 3433.015 | 3351.968 | 0.02 | 0.02 | 0.125 / 0.141 |
| Genetic test results (PV, N, UN) & genetic test result * agreement |  |  |  | X | 3453.644 | 3347.659 | 0.36 | 0.02 | 0.131 / 0.146 |
| **PAHC domain: Familial issues** |  |  |  |  |  |  |  |  |  |
| **Variables** |  |  |  |  |  |  |  |  |  |
| Samples | X | X | X | X | 3410.924 | 3373.553 | - | 0.02 | 0.053 / 0.074 |
| Agreement |  | X | X | X | 3370.070 | 3314.014 | <0.0000 | 0.02 | 0.156 / 0.177 |
| Time T1 to T2 & Time * agreement |  |  | X | X | 3385.364 | 3304.394 | 0.05 | 0.02 | 0.169 / 0.186 |
| Genetic test results (PV, N, UN) & genetic test result * agreement |  |  |  | X | 3407.227 | 3301.343 | 0.55 | 0.03 | 0.176 / 0.202 |
| **PAHC domain: Emotions** |  |  |  |  |  |  |  |  |  |
| **Variables** |  |  |  |  |  |  |  |  |  |
| Samples | X | X | X | X | 3416.982 | 3379.599 | - | 0.02 | 0.052 / 0.073 |
| Agreement |  | X | X | X | 3337.243 | 3281.169 | <0.0000 | 0.01 | 0.214 / 0.222 |
| Time T1 to T2 & Time * agreement |  |  | X | X | 3351.479 | 3270.483 | 0.03 | 0.01 | 0.229 / 0.236 |
| Genetic test results (PV, N, UN) & test result * agreement |  |  |  | X | 3374.618 | 3268.700 | 0.78 | 0.01 | 0.230 / 0.240 |
| **PAHC domain: Living with cancer** |  |  |  |  |  |  |  |  |  |
| **Variables** |  |  |  |  |  |  |  |  |  |
| Samples | X | X | X | X | 3404.810 | 3367.451 | - | 0.02 | 0.054 / 0.074 |
| Agreement |  | X | X | X | 3416.168 | 3360.129 | 0.06 | 0.02 | 0.067 / 0.088 |
| Time T1 to T2 & Time * agreement |  |  | X | X | 3436.376 | 3355.431 | 0.32 | 0.02 | 0.074 / 0.090 |
| Genetic test results (PV, N, UN) & genetic test result * agreement |  |  |  | X | 3457.060 | 3351.208 | 0.38 | 0.03 | 0.083 / 0.109 |
| **PAHC domain: Children-related issues** |  |  |  |  |  |  |  |  |  |
| **Variables** |  |  |  |  |  |  |  |  |  |
| Samples | X | X | X | X | 2465.947 | 2430.614 | - | 0.03 | 0.057 / 0.081 |
| Agreement |  | X | X | X | 2455.333 | 2402.333 | <0.0000 | 0.03 | 0.127 / 0.154 |
| Time T1 to T2 & Time * agreement |  |  | X | X | 2473.420 | 2396.865 | 0.24 | 0.02 | 0.137 / 0.152 |
| Genetic test results (PV, N, UN) & genetic test result * agreement |  |  |  | X | 2488.696 | 2388.585 | 0.08 | 0.04 | 0.157 / 0.194 |

| *PV=pathogenic variant, N=negative, UN=uninformative. For distress at T2, the basic model with the intercept, random effect of clinicians on the intercept, and the fixed effects of samples is compared to is compared to: 1) a first model as above, 2) a second model including length of time and its interaction with agreement, and 3) a third model including test results and their interaction with agreement. The best model is selected based on Bayesian Information Criterion (BIC) estimates. Marginal R2 provides the variance explained only by fixed effects and conditional R2 provides the variance explained by the entire model, i.e., both fixed effects and random effects. Intra-class correlation coefficient (ICC) informs on the clinician variability effect. Statistical significance tests take ‘True negative’ as the reference category. *, ***=p values < 0.05; 0.001. |
| --- |

| **Supplementary material table S7. Estimates for distress after the post-test (T2) consultation according to mixed linear models including the genetic test result, samples, HADS at T1, time lapse between T1 and T2 (all samples) and personal breast cancer (samples 1 & 2)** |
| --- |

| **Model:** | **Basic** | **1** | **2** | **3** | **BIC** | **Deviance** | **p value** | **ICC** | **Marginal R2/Conditional R2** |
| --- | --- | --- | --- | --- | --- | --- | --- | --- | --- |
| **Outcome: HADS at T2 (samples 1 & 2)** |  |  |  |  |  |  |  |  |  |
| **Variables** |  |  |  |  |  |  |  |  |  |
| Samples | X | X | X | X | 1503.554 | 1481.854 | - | 0.02 | 0.020 / 0.035 |
| HADS at T1 & Personal breast cancer |  | X | X | X | 1303.642 | 1271.092 | <0.0000 | 0.01 | 0.609 / 0.613 |
| Time T1 to T2 |  |  | X | X | 1308.140 | 1270.165 | 0.34 | 0.01 | 0.610 / 0.614 |
| Genetic test results (PV, N, UN, VUS) |  |  |  | X | 1319.667 | 1265.418 | 0.19 | 0.02 | 0.614 / 0.620 |
| **Outcome: HADS at T2 (samples 3 & 4)** |  |  |  |  |  |  |  |  |  |
| **Variables** |  |  |  |  |  |  |  |  |  |
| Samples | X | X | X | X | 2114.391 | 2091.432 | - | 0.03 | 0.041 / 0.070 |
| HADS at T1 |  | X | X | X | 1861.970 | 1833.271 | <0.0000 | 0.02 | 0.577 / 0.587 |
| Time T1 to T2 & Genetic test result (PV, N, UN) |  |  | X | X | 1867.602 | 1833.163 | 0.74 | 0.04 | 0.575 / 0.590 |

| *PV=pathogenic variant, N=negative, UN=uninformative negative, VUS=variant of uncertain significance. VUS not communicated to counselees in samples 3 & 4. |
| --- |

**Supplementary material table S8. Beta coefficients (standard error) and HADS predicted means (95% confidence interval) for distress after the post-test (T2) consultations according to the genetic test result.^1^**

|  | **Sample 1 - FR & Sample 2 - SP** | | **Sample 3 - FR & Sample 4 - GE** | |
| --- | --- | --- | --- | --- |
| **PREDICTORS** | **Predictors' B (standard error)** | **HADS predicted means**  **(95% CI)** | **Predictors' B (standard error)** | **HADS predicted means**  **(95% CI)** |
| ***Test result*** |  |  |  |  |
| Uninformative | REF | 10.8 (10.0-12.0) | REF | 10.0 (8.4-10.9) |
| Pathogenic variant | -0.75 (0.92) | 10.1 (8.5-11.6) | -0.88 (0.86) | 8.3 (7.2-9.4) |
| Negative | -1.97 (1.27) | 8.8 (6.9-10.8) | -1.35 (0.90) | 8.8 (7.5-10.1) |
| VUS | -1.45 (0.89) | 9.4 (7.5-11.2) | - | - |

Note: FR=France; GE=Germany; SP=Spain. T1=within 1 month after the pre-test consultation; T2=within 3 months after the post-test consultation.

**^1^** Beta coefficients and HADS predicted means from mixed linear models including the random effect of clinicians, samples, HADS at T1, time lapse between T1 and T2, and genetic test results (all samples), and personal breast cancer (samples 1 & 2). Best models includes samples, HADS at T1 (all samples) and personal breast cancer (samples 1 & 2)
